# Systematic review and meta-analysis of knowledge on PMTCT of HIV/AIDS and Association factors among reproductive age women in Ethiopia, 2022

**DOI:** 10.1186/s12879-023-08461-z

**Published:** 2023-07-25

**Authors:** Sisay Yitayih Kassie, Alex Ayenew Chereka, Yitayish Damtie

**Affiliations:** 1 Department of Health Informatics, College of Health Science, Mettu University, P.O.Box: 318, Mettu, Ethiopia; 2 Department of public health, college of medicine and health science, Injibara University, Injibara, Ethiopia

**Keywords:** Knowledge, PMTCT reproductive age women, Ethiopia

## Abstract

**Background:**

Despite increased interventions implemented for the prevention of mother-to-child transmission of HIV, There is still a vertical transmission. Hence, this study tried to assess the pooled prevalence of knowledge on PMTCT and factors associated with residence, ANC follow-up, and knowledge about HIV/AIDS among reproductive age women in Ethiopia.

**Methods:**

The Preferred Reporting Items for Systematic Reviews and Meta-Analyses (PRISMA) guideline was followed to review either published or unpublished studies in Ethiopia regarding knowledge on PMTCT. A comprehensive search of international databases, including Google Scholar, Cochrane Library, PubMed, HINARI, Embrace, Web of Science, Scopus, ProQuest, CINAHL, and Global Health, were searched. The data were analyzed using STATA/SE version 14. The random-effect model was used to estimate the effect size, and I-squared statistics and Egger’s test were used to assess the heterogeneity and publication bias, respectively.

**Results:**

14 out of 14,091 assessed articles met inclusion criteria and were included in the analysis. The estimated pooled level of knowledge on PMTCT among reproductive-age groups was 62.15% [(95% CI: 43.63–80.66)]. Residence [(OR = 4.8, 95%CI: 2.99, 7.85)], ANC follow-up [(OR = 4.2, 95%CI: 2.21, 7.98)], and having sufficient knowledge about the diseases [(OR = 4.9, 95% CI: 3.6, 6.66)] were found to be significant predictors of knowledge on PMTCT among reproductive-age groups.

**Conclusion:**

Strategies to improve the knowledge of PMTCT in Ethiopia should focus on rural women, improving knowledge about HIV/AIDS, and ANC follow-up. Efforts are also needed to involve husbands and related organization in the prevention of mother to child transmission of HIV.

**Supplementary Information:**

The online version contains supplementary material available at 10.1186/s12879-023-08461-z.

## Background

Mother-to-child transmission occurred during pregnancy, labor, delivery, and breastfeeding [1–4]. More than 90% of children with HIV are believed to have the disease through mother-to-child transmission (MTCT). If the mother does not nurse the infant, the rate of MTCT without particular therapy ranges from 15 to 30%. Breastfeeding in the second year of life was believed to increase the rate of infection by 45% [5, 6]. Despite several efforts that have been made to prevent human immunodeficiency virus (HIV/AIDS), the pandemic still continues to seriously threaten worldwide public health concerns [7]. Globally, 38.4 million people were living with HIV, 1.5 million became newly infected, and 1.7 million were children in 2021[8]. About 68% of them were from sub-Saharan African (SSA) countries, of which 2.3 million were newly infected [9, 10]. In Ethiopia, around 612,925 people lived with HIV (PLHIV), with an estimated HIV prevalence of 0.96%. The highest HIV prevalence rates were found in Addis Ababa (3.4%) and the Gambella region (4.8%), according to the 2016 Ethiopian Demographic and Health Survey (EDHS) report. The rate of prevalence varies by demographic characteristics, region, and population group. Women (1.2%) have a greater HIV prevalence than men (0.6%), and urban HIV prevalence (2.9%) is seven times higher than rural HIV prevalence (0.4%) [11–13].

Comparably, in the last decade of years, mortality and morbidity due to HIV/AIDS declined due to preventive initiatives such as ART use and the prevention of mother-to-child transmission. The prevention of mother-to-child transmission (PMTCT) program saves nearly 1.4 million new childhood HIV infections and is a major contributor to the elimination of new HIV infections in low- and middle-income countries [14, 15]. PMTCT is a fundamental approach to combating HIV/AIDS. In 2013, Ethiopia implemented Option B+, and all HIV/AIDS-infected pregnant women received triple ARV (anti-retroviral) drugs without initial CD4 testing [16, 17].

Although a remarkable achievement has been made and new HIV infections among children have declined, HIV remains the major cause of child morbidity and mortality in low resource countries and continues to be a major public health threat, especially for children less than 5 years old [18, 19]. Evidence showed that, this rate of transmission is reduced with effective intervention [20]. Even though there are still many unresolved barriers or challenges to the program, particularly in sub-Saharan Africa. Among the main barriers, low knowledge of PMTCT is commonly mentioned [21–29]. The knowledge of reproductive-age women on the prevention of mother-to-child transmission (MTCT) of HIV plays a crucial role in limiting the number of children with HIV/AIDS [19, 30, 31]. Timely interventions like testing for HIV during pregnancy and delivery, using preventive antiretroviral (ARV) drugs, and improving infant feeding practices help to minimize the risk of a child getting HIV infection. Not only this, but also knowledge about PMTCT plays a great role in protecting themselves, their husbands, and their children from HIV infection [27, 32].

Knowledge of PMTCT was assessed among reproductive-age women in different parts of Ethiopia [1, 9, 16, 25–27, 29, 33–36]. However, systematic reviews and meta-analysis were not assessed in Ethiopia. According to various studies done elsewhere, knowledge of PMTCT of HIV/AIDS is correlated with factors such as maternal age, maternal education, wealth status, occupation, marital status, media exposure, and residence [7, 9, 14, 16, 21, 22, 25, 26, 33, 37–41].

There is no national representative estimate of the pooled level of knowledge on PMTCT and associated factors in Ethiopia. So, this study aimed to estimate the pooled level of knowledge on PMTCT and its association with antenatal care follow-up (ANC follow-up) and place of residence and knowledge about HIV in Ethiopia. ANC follow-up and knowledge about HIV are clinically important and frequently mentioned factors affecting knowledge of PMTCT. However, previous literature has inconsistent findings across the included articles in this meta-analysis. Since the prevention of HIV/AIDS from mother-to-child is one of the Sustainable Development Goals (SDGs). To prove evidence to support the program, this meta-analysis will generate crucial evidence for program planners and policymakers to design evidence-based interventions to increase the prevention of mother-to-child transmission. Furthermore, this evidence provides an evidence that helps program managers and policymakers to evaluate their interventions to fight the pandemic.

## Methods

### Searching strategy

This meta-analysis followed the Preferred Reporting Items for Systematic Reviews and Meta-Analyses (PRISMA-2020) guideline [42]. A comprehensive search of international databases including CINAHL, Google Scholar, Cochrane Library, PubMed, HINARI, Embrace, Web of Science, Scopus, ProQuest, Ovid, EBSCOhost, and Global Health, was carried out to estimate the pooled prevalence of knowledge on PMTCT and its association with place of residence, ANC follow-up, and sufficient knowledge on HIV/AIDS among reproductive-age groups in Ethiopia.

The search for the study articles was conducted from December 1 through December 315, 2022, independently by two reviewers (YD and SY) and articles published or released from 2000 to December 15, 2022 were included in this systematic review and meta-analysis. A systematic searching strategy with a combination of the following terms was used to find the published and released articles as a blueprint. In the beginning, we have employed (“Knowledge”) AND (“prevention of mother-to-child transmission”) OR (“PMTCT”) OR (“prevention”) AND (“MTCT”) AND (“pregnant mothers” “lactating mothers”) AND (“HIV positive mothers”) OR (“men”) AND (“Ethiopia”) search strategy.

In addition, studies were identified using the following key terms: “assessment”, “prevalence”, “proportion”, “level”, “knowledge”, “PMTCT”,“ prevention of mother-to-child transmission”, “MTCT”, ANC follow-up”, PNC follow-up”, “predictors”, “determinants”, “factors”, “associated factors”, “women”, “husbands”, “lactating mothers”, " HIV positive women”, “reproductive age groups”, “Ethiopia” using the Boolean operators “AND” and “OR”.

PubMed.

(((((((((knowledge [(All Fields]) OR PMTCT[(All Fields])) OR HIV [(All Fields])) OR [(All Fields])) AND (“HIV/AIDS”[(MeSH Terms]) OR knowledge of PMTCT[(Text Word]))) OR (“knowledge of PMTCT”[(MeSH Terms]) OR PMTCT about HIV/AIDS [(Text Word]))) AND (“reproductive age women”[(MeSH Terms]) OR mothers[(Text Word])))

Humans, from 2000/1/12–2022/31/12.

HINARI.

((((((“assessment”, “prevalence”, “proportion”, “level”, “knowledge”, “PMTCT”,“ prevention of mother-to-child transmission”, “MTCT”, ANC follow-up”, PNC follow-up”, “predictors”, “determinants”, “factors”)))))), “associated factors”, “women”, “husbands”, “lactating mothers”, " HIV positive women”, “reproductive age groups”, (“Ethiopia”).

Filter applied, Humans, from 1/1/2000- 12/31/2022.

### Inclusion and exclusion criteria

All observational studies (cross-sectional, case-control, and cohort studies), studies conducted in Ethiopia, published from 2000 up to early December, 2022, articles published and unpublished studies of reproductive-age women, articles reporting a good or poor level of knowledge of PMTCT about HIV/AIDS, studies with full text, and studies reporting in English were included in the systematic review and meta-analysis. Whereas, studies without full text, fully qualitative studies, or that did not assess the knowledge of PMTCT about HIV/AIDS were excluded.

### Outcome measurement

This meta-analysis measured two key outcomes. The primary outcome of the study was to estimate the pooled prevalence of knowledge on PMTCT about HIV/AIDS. Which was computed from the individual articles by dividing the number of reproductive age groups of individuals who have good knowledge by the total sample size multiplied by 100. Scholars have used different measurement scales to determine knowledge of PMTCT. Some literatures have been used five item multiple choice questions with a correct score of 1 point and a wrong response a score of 0 point. The responses were summed, and the mean score value was calculated. The score above or equal to the mean value was categorized as knowledgeable on PMTCT and below the mean value was considered as not knowledgeable [43]. Another’s have built three item knowledge index question (using antiretroviral therapy (ART) drugs, safe delivery, only breastfeeding up to 6 months, and there are special medicines that a doctor or a nurse can give to a woman infected with HIV to reduce the risk of transmission to the baby with yes/no responses); then, the index was categorized as not full knowledge (score < 3) and full knowledge (score = 3). The second outcome was the association factors. Respondents who have antenatal care follow-up with a response of yes/no, residence, and having sufficient or comparable knowledge about HIV/AIDS, which was measured with 13 questions: four questions on knowledge of HIV prevention, four questions on knowledge of HIV transmission, and five on misconceptions about modes of HIV transmission. Based on the responses to these knowledge questions, the index was categorized as insufficient knowledge (score ≤ 6) and sufficient knowledge (score 7–13).

### Data extraction and quality assessment

All articles retrieved from different databases were exported to the endnote reference manager, where duplicates were identified and removed. The remaining articles were screened based on their titles and abstracts and evaluated in the context of the inclusion criteria by three independent reviewers (SY and YD). Then the Joanna Briggs Institution (JBI) quality assessment tool was used to appraise the quality of the screened articles, and those articles scoring 50% and above were included in the analysis [44, 45]. In this meta-analysis, all included studies scored 50% and/or above. Thus, all are included in this systematic review and meta-analysis. Two authors (YD and SY) independently assessed the quality of the studies and the mean score was taken to manage the different results obtained from both reviewers.

All necessary data were extracted using a Microsoft Excel sheet. The data extraction sheet includes the name of the author, study area, region, publication year, year of study, study design, study area, level of knowledge of PMTCT, response rate, sample size, study population, place of residence, antenatal care follow-up, and having comprehensive knowledge about the disease in the form of two by two tables. Three independent authors (YD and SY) extracted all data, and the discrepancy between reviewers was resolved through consensus.

### Data analysis

All extracted data were exported to STATA version 14 for further analysis. The random effect model with a *p*-value < 0.05 was used to compute the pooled prevalence of knowledge of PMTCT among reproductive age groups in Ethiopia. In addition, the association between residence, ANC follow-up, and knowledge of HIV was statistically estimated using pooled odds ratios with 95% CI. The I^2^ statistic was used to assess the heterogeneity between the included studies, and I^2^ tests with a value of 25%, 50%, and 75% were considered to have low, medium, and high heterogeneity, respectively. Subgroup analysis and univariate meta-regression were carried out to identify the source of variation among studies that exhibited severe heterogeneity. Moreover, publication bias was assessed using the funnel plot and Egger’s test. A p-value of less than 0.05 in the Egger regression test is considered to indicate the presence of statistically significant publication bias.

## Result

### Study selection

**Fig. 1:**
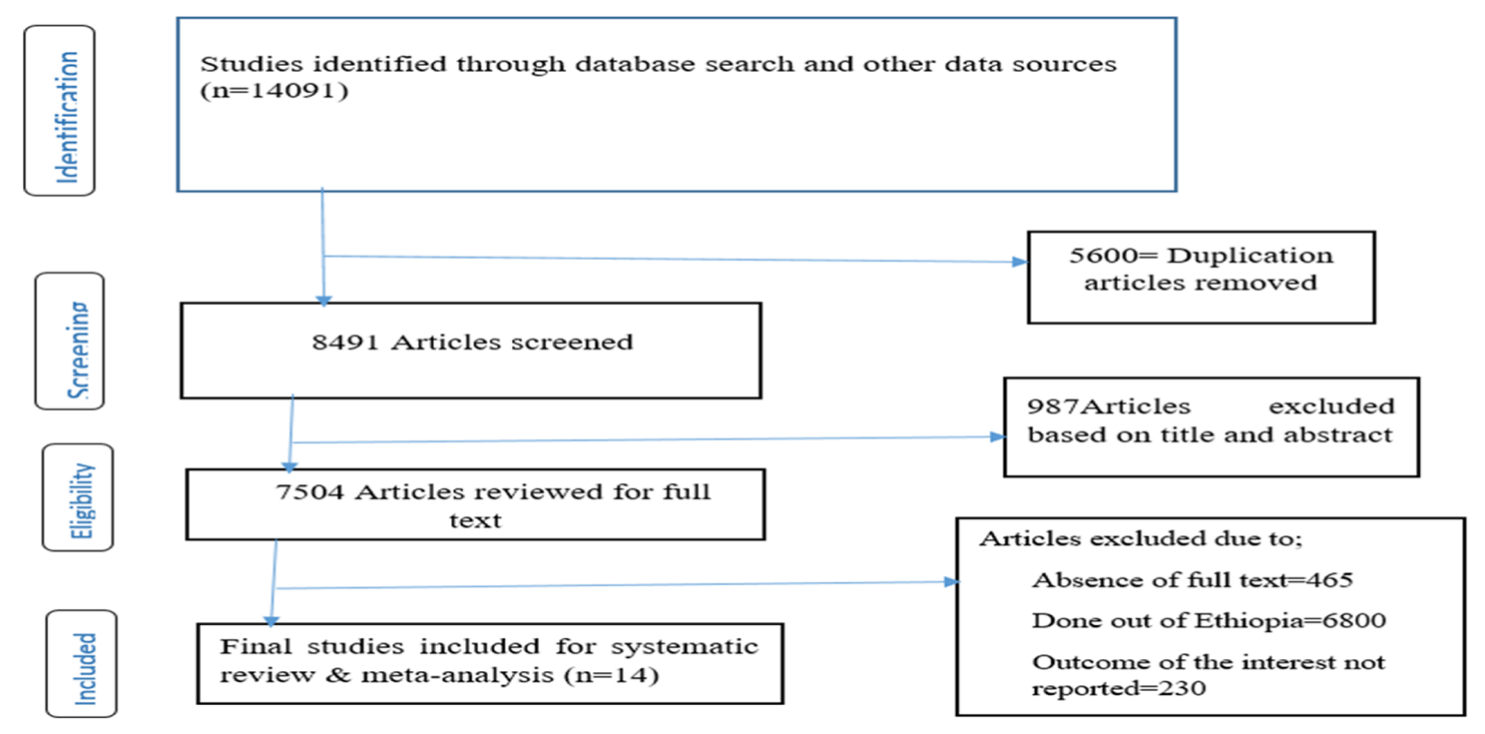
PRISMA flow diagram describing the selection of studies for systematic review and meta-analysis in Ethiopia, 2022

A total of 14,091 articles were identified by searching the databases: PubMed, CINAHL, Google Scholar, the Cochrane Library, HINARI, and Global Health. Among this, 5600 articles were removed using endnote referencing software due to duplication, 987 articles were dropped due to their titles and abstracts, and the remaining 7504 articles were critically appraised based on the inclusion and exclusion criteria. Finally, 14 full-text articles were included in the systematic review and meta-analysis (see Fig. 1).

### Characteristics of the included studies

14 studies with 4848 respondents were included to estimate the pooled level of knowledge on PMTCT about HIV/AIDS among reproductive-age women in Ethiopia and associated factors in this systematic review and meta-analysis. Seven studies were carried out in the Amhara region, three were conducted in the SNNPR, two were in the Oromia region, and the rest were in the Bishangul Gumuz region and Addis Ababa. Almost all the included studies used facility-based method and only one used a community-based cross-sectional study design (see Table 1).

**Table 1:** Characteristics of individual studies done on knowledge of PMTCT of HIV/AIDS among reproductive age groups in Ethiopia, 2022

Authors & study year	Region	Study year	Sample size	Population	Prevalence	Quality score
Tesfaye et al.2014	Oromia	2014	238	pregnant mothers	88.1	76%
Amanuel Abajobir.2012	SNNPR	2012	238	pregnant mothers	82.3	84%
Abtew.s et al. 2014	Beneshangul Gumuz	2014	386	pregnant mothers	17.4	65%
Tsegaye. D et al. 2015	Amhara	2015	190	Both Pregnant and lactating mothers	72.1	70%
Jebessa.S et al. 2004	AA	2004	319	Lactating mothers	76.8	86%
Liyeh et al. 2016	Amhara	2016	853	Reproductive age women	22.4	80%
Hailu D, et al. 2017	SNNPR	2017	170	pregnant mothers	71.2	75%
Alemu. Y et al. 201	Amhara	2012	416	pregnant mothers	52	77%
Malaju et al. 2011	Amhara	2011	400	pregnant mothers	83.5	90%
Abebe et al. 2017	Amhara	2017	125	pregnant mothers	61	92%
Kahsay 2015	Amhara	2015	297	pregnant mothers	55.8	73%
Tekelia.D 2017	Amhara	2017	402	HIV + women	68.91	80%
Tigabu. W et al. 2015	SNNPR	2015	224	pregnant mothers	83.5	80%
Dina. G et al. 2019	Oromia	2019	590	HIV + women	30.7	87%

### Prevalence of knowledge about PMTCT among reproductive age women in Ethiopia

**Fig. 2:**
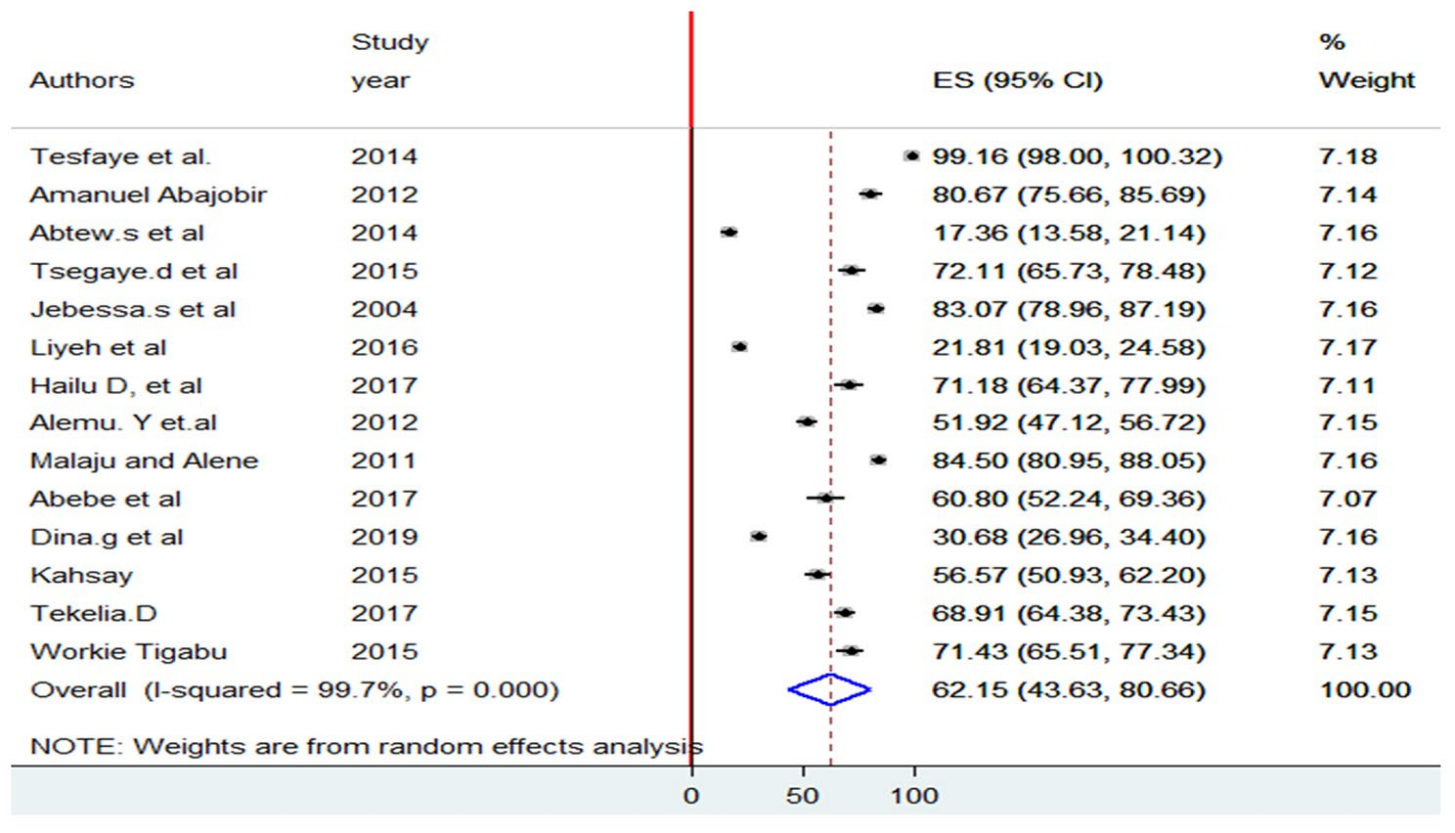
Forest plot diagram indicates pooled level of knowledge on PMTCT about HIV/AIDS among reproductive age women in Ethiopia, 2022

The pooled level of knowledge of PMTCT among reproductive age women in Ethiopia was 62.15% (95% CI: 43.63, 80.66). A random-effects model was employed to estimate the pooled effect due to significant heterogeneity across the included studies (I^2^ = 99.7%, p = 0.000). Therefore, this result suggests that there is significant heterogeneity across the primary studies and needs subgroup analysis (see Fig. 2).

### Publication Bias

**Fig. 3:**
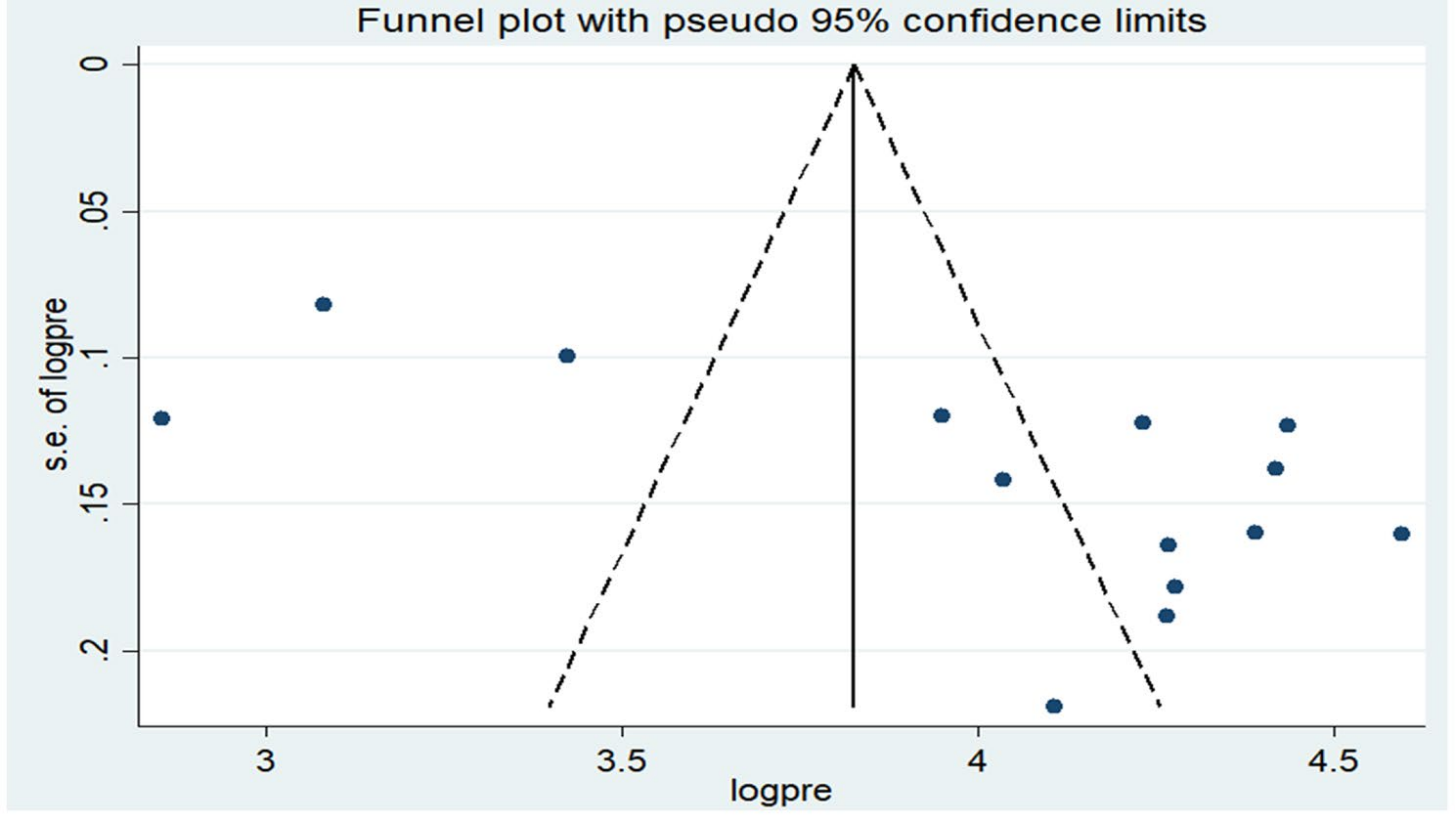
Funnel plot of the pooled prevalence of knowledge about PMTCT among reproductive age women in Ethiopia, 2022

To identify the presence or absence of publication bias, both the funnel plot and the Eggers test were employed. The result of the funnel plot indicates an asymmetrical distribution, which is a sign of the presence of publication bias (Fig. 3). However, the result of the Eggers test indicates the absence of publication bias (P = 0.21).

### Subgroup analysis and Meta-regression

To identify the source of heterogeneity among the included studies, subgroup analysis was performed based on the region in which the studies were conducted, sample size, and study years. In this analysis, the prevalence of knowledge on PMTCT was significantly higher among studies done in the SNNPR region [27, 36, 41] followed by studies conducted in Amhara and other regions respectively. In addition, the lowest pooled level of knowledge on PMTCT was observed among studies conducted with a sample size of above 400 (see Table 2).

**Table 2:** Subgroup prevalence of knowledge about PMTCT among reproductive age women in Ethiopia, 2022(n = 14)

Variables	Characteristics	Included studies	Estimate (95% CI)	I^2^
Region	Amhara	7	59.49 (38.78, 80.20)	99.3%
	SNNPR	3	74.69 (68.13, 81.24)	73.2%
	Others	4	57.58(14.10, 101.07)	99.9%
Sample size	<=400	10	69.71(51.59, 87.83)	99.5%
	> 400	4	43.27(22.48, 64.06)	99.2%
Study years	<=2015	9	68.55(48.91, 88.18)	99.6%
	> 2015	5	50.53(29.34, 71.73)	99.1%

SNNPR-South nation and nationalities of people region, others- Addis Ababa, Bishangul Gumuz, and Oromia

### Univariate and meta-regression analysis

A univariate meta-regression analysis was done using publication year and response rate as predictor variables, which indicates that publication year was found to be a statistically significant source of heterogeneity among the included articles (see Table 3).

**Table 3:** Univariate meta-regression analysis to identify factors associated with the heterogeneity of knowledge about PMTCT among reproductive age women in Ethiopia, 2022

Variables	Coefficient	*P*-value
Publication year	-5.190158	0.017
Response rate	2.612247	0.164

### Factors associated with knowledge of PMTCT about HIV/AIDS

**Fig. 4:**
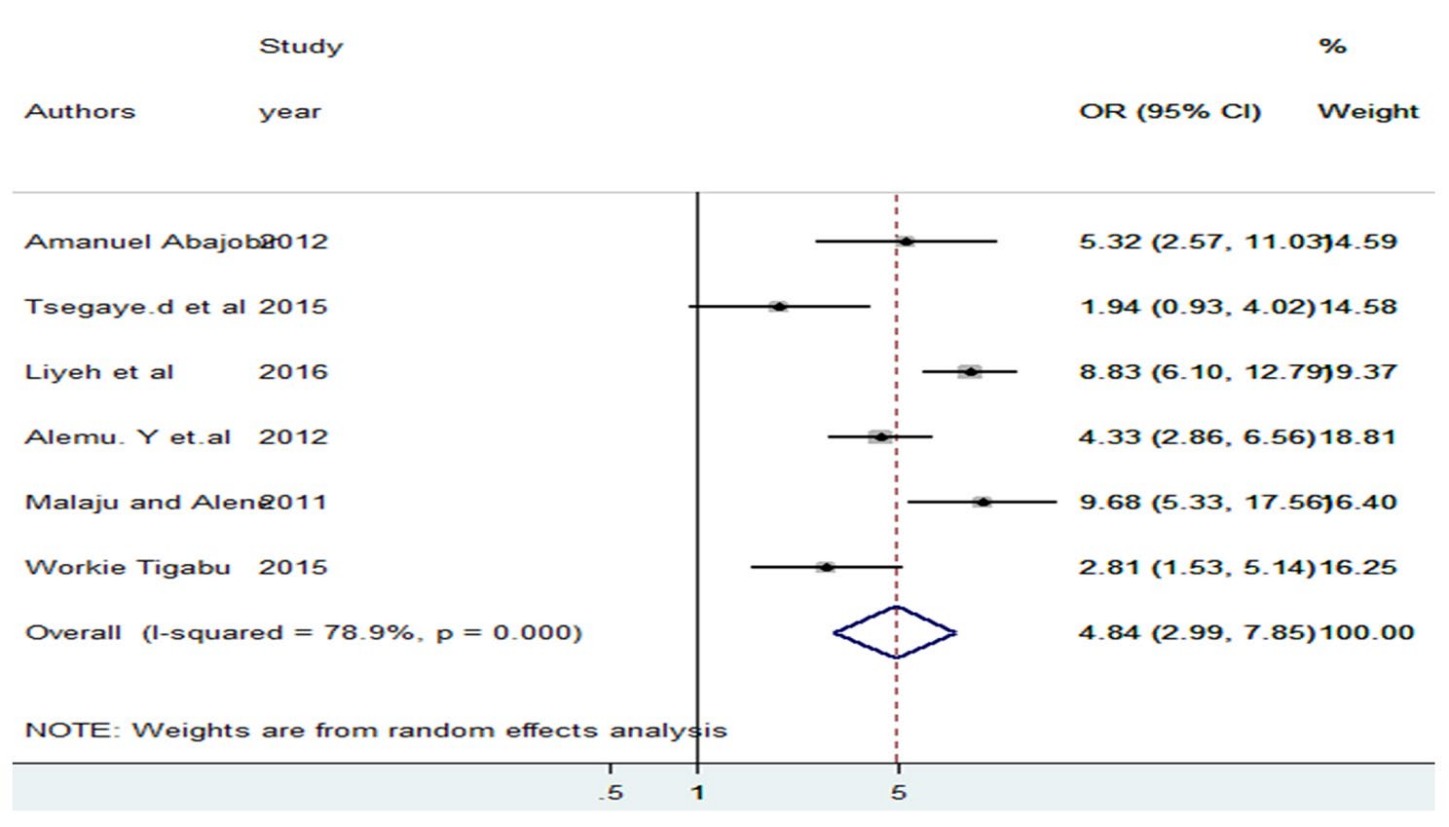
The pooled odds ratio of places of residence among reproductive age women in Ethiopia, 2022

The association between residence and knowledge of PMTCT was assessed using six studies. In this analysis, study participants who have lived in urban areas were 4.84 (2.99, 7.85) times more likely to have good knowledge of PMTCT about HIV/AIDS compared to their counterparts. A random fixed effects meta-analysis model was employed to examine the association between residence and knowledge of PMTCT about HIV/AIDS due to the presence of heterogeneity (I^2^ = 78.9%, p = 0.000). In addition, Eggers’s test was used to assess publication bias and indicated an absence of publication bias (p = 0.287) (see Fig. 4).

**Fig. 5:**
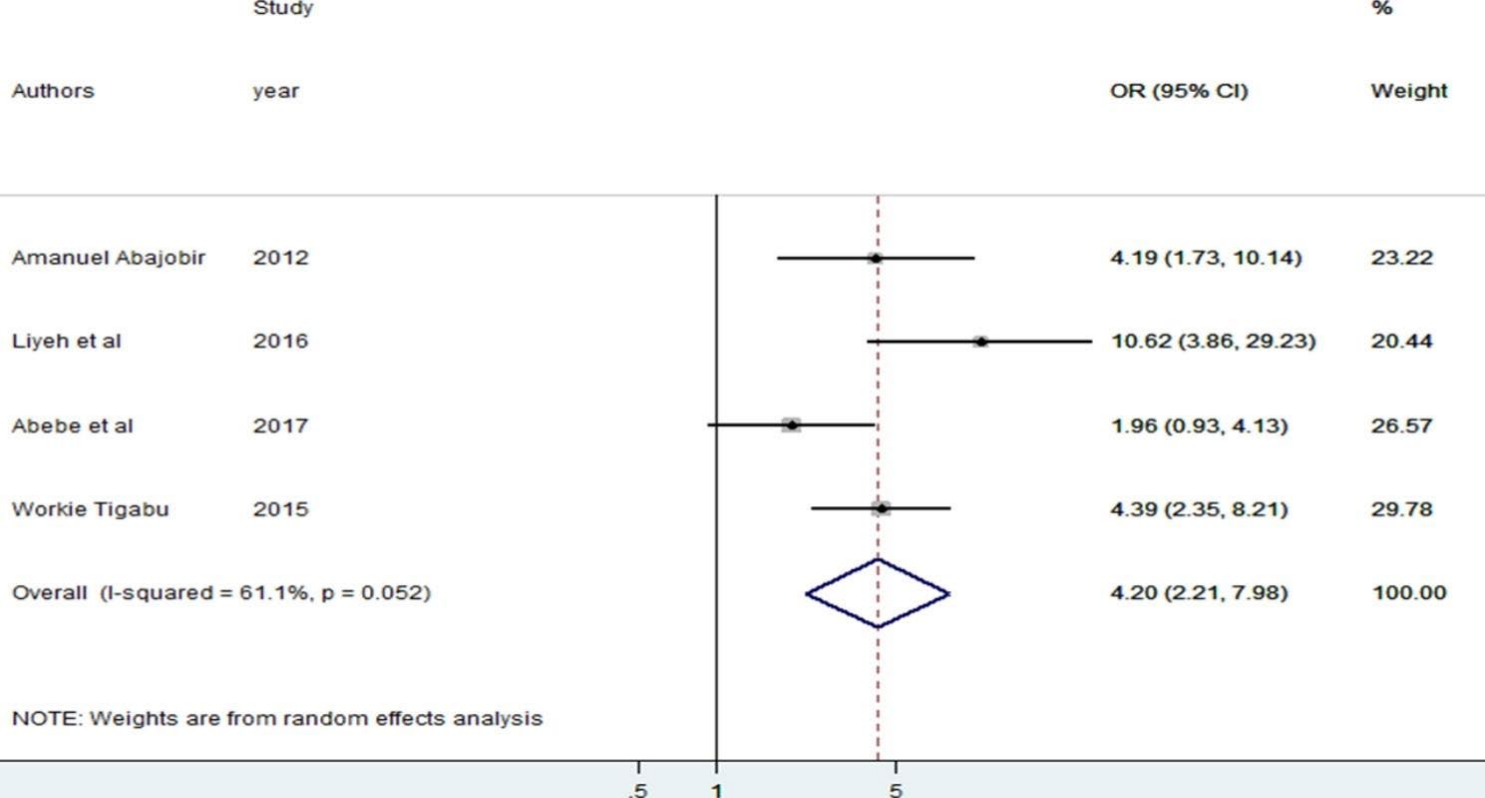
The pooled odds ratio of antenatal care follow-up among reproductive age women in Ethiopia, 2022

Four studies were used to assess the statistical association between antenatal care visits and knowledge of PMTCT. A statistical association was observed in three included articles, and a non-association was observed in one study. Study subjects who had antenatal care follow-up were 4.20 (OR = 4.20, 2.21, 7.98) times more likely to have knowledge of PMTCT about HIV/AIDS than those who did not have antenatal care follow-up. The random fixed effects analysis indicates no heterogeneity has been invited by the included studies (I^2^ = 61.1%, p = 0.052). In addition, Egger’s test result shows no publication bias (p = 0.527) (see Fig. 5).

**Fig. 6:**
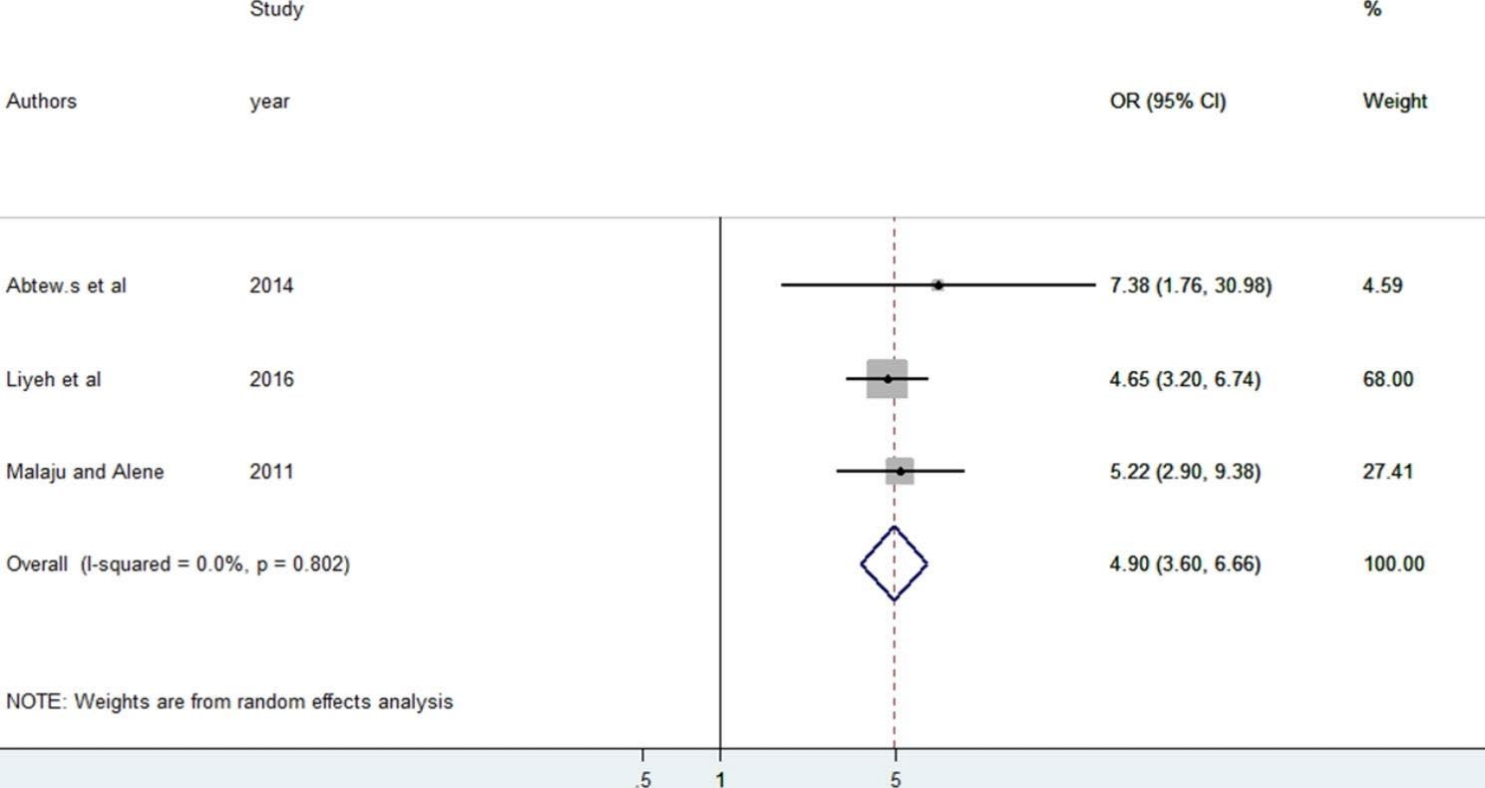
The pooled odds ratio of HIV/AIDS knowledge among reproductive age women in Ethiopia, 2022

Finally, three studies were used to identify the association between comprehensive knowledge of HIV/AIDS and knowledge of PMTCT. The result indicates an association has been observed among the included studies and reveals that study subjects who have comprehensive knowledge about the disease were 4.9 (95% CI: 3.60, 6.66) times more likely to have knowledge about PMTCT than their counterparts. The results of the random fixed effect meta-analysis revealed no significant heterogeneity across the included studies (I2 = 0.0%, p = 0.802). Publication bias was also assessed using Egger’s test, which indicated no presence of publication bias in the included studies (P = 0.059) (see Fig. 6).

## Discussion

The purpose of this systematic review and meta-analysis is to give up-to-date and current information on the pooled level of knowledge on PMTCT among reproductive-age women in Ethiopia. Having comprehensive knowledge on PMTCT among women and husbands, who play a major role in transmitting the disease to their children, helps implement policies and strategies like ARV prophylaxis for children who will be born to HIV-exposed mothers, initiating ART drugs for mothers based on option B + rules, and preventing extended pregnancies. The pooled level of knowledge on PMTCT of HIV/AIDS among study participants in Ethiopia was 62.15 (95% CI: 43.63, 80.66). This finding is comparable to studies done in India, 77%[4] but lower than studies done in Nepal 86.3% [40]. The possible justification for the discrepancy could be due to a lack of awareness and providing in-service counseling by healthcare providers. Another possible reason might be due to a lack of media coverage regarding the disease pandemic and possible methods of preventing the transmission of the disease from mother to child.

The association between residence, ANC follow-up, and knowledge on PMTCT about HIV/AIDS was assessed. Study subjects who were in urban residences had 4.84 times more knowledge on PMTCT about HIV/AIDS. This finding is consistent with studies done in Ethiopia [20, 26, 41]. In addition, individuals who followed antenatal care were 4.20 times more likely to have knowledge on PMTCT about HIV/AIDS compared to their counterparts. This finding is nearly comparable with a study finding done in Ethiopia [26, 41].

In this meta-analysis, ANC follow-up was found to be a statistically significant predictor of knowledge of PMTCT. Women who had ANC visits were more likely to have a good level of knowledge on PMTCT. The possible reason could be that woman who attend ANC follow-up can access PMTCT information from their care providers during counseling. Having sufficient knowledge about HIV/AIDS was positively correlated with knowledge of PMTCT. Individuals who have sufficient knowledge about the disease were 4.9 times more likely to have good knowledge on PMTCT. This finding is comparable with the study done in Ethiopia [20]. The possible reason might be that participants who had sufficient knowledge about HIV/AIDS could get knowledge about PMTCT from their healthcare providers during service utilization, from mass media, counseling, and be able to seek PMTCT information from digital technology. This possible explanation was supported by the study done in Ethiopia [39].

## Conclusions and recommendations

In Ethiopia, the overall level of knowledge on PMTCT about HIV/AIDS among the reproductive-age group was poor. Residence, good knowledge about the disease, and ANC follow-up were found to be significant predictor of knowledge on PMTCT. As a result, policymakers and program managers should focus their efforts on promoting women to have ANC follow-up and developing interventional strategies for increasing their knowledge about the pandemic and strengthening awareness of PMTCT in the rural community.

### Strengths and limitations

Despite its strength, which provides crucial evidence for the knowledge of PMTCT and its factors among reproductive-age women, it has a limitation. The limitation of this research is the relatively small number of articles included in this analysis and the sample size of the included articles used, which will affect the overall point estimates of the knowledge on PMTCT. In addition, the heterogeneity of this study still existed even after a final random effect model analysis.

## Electronic supplementary material

Below is the link to the electronic supplementary material.

Supplementary Material 1

## Data Availability

The datasets used and/or analyzed during the current study available from the corresponding author on reasonable request.

## List of abbreviations

ANC: Antenatal care
ART: Antiretroviral therapy
ARV: Antiretroviral prophylaxis
EDHS: Ethiopian Demographic and health survey
HIV/AIDS: Human Immune Virus/ Acquired Immunodeficiency Syndrome
JBI: Joana Brigg’s Institute
MTCT: Mother to child transmission
PMTCT: Prevention of mother to child transmission
PRISMA: Preferred Reporting Items for Systematic Reviews and Meta:Analysis
SDG: Sustainable Development Goal
SSA: Sub:Saharan African
SNNPR: South nation nationality and people region
WHO: World Health Organization

## Declarations

### Patient and public involvement

No patients and public involved in this study setting.
